# Supplementary figures and images for: Isolating selective from non-selective forces using site frequency ratios
Source: PLoS Genet. 2025 Apr 21;21(4):e1011427. doi: 10.1371/journal.pgen.1011427 (PMC12064048; doi:10.1371/journal.pgen.1011427)

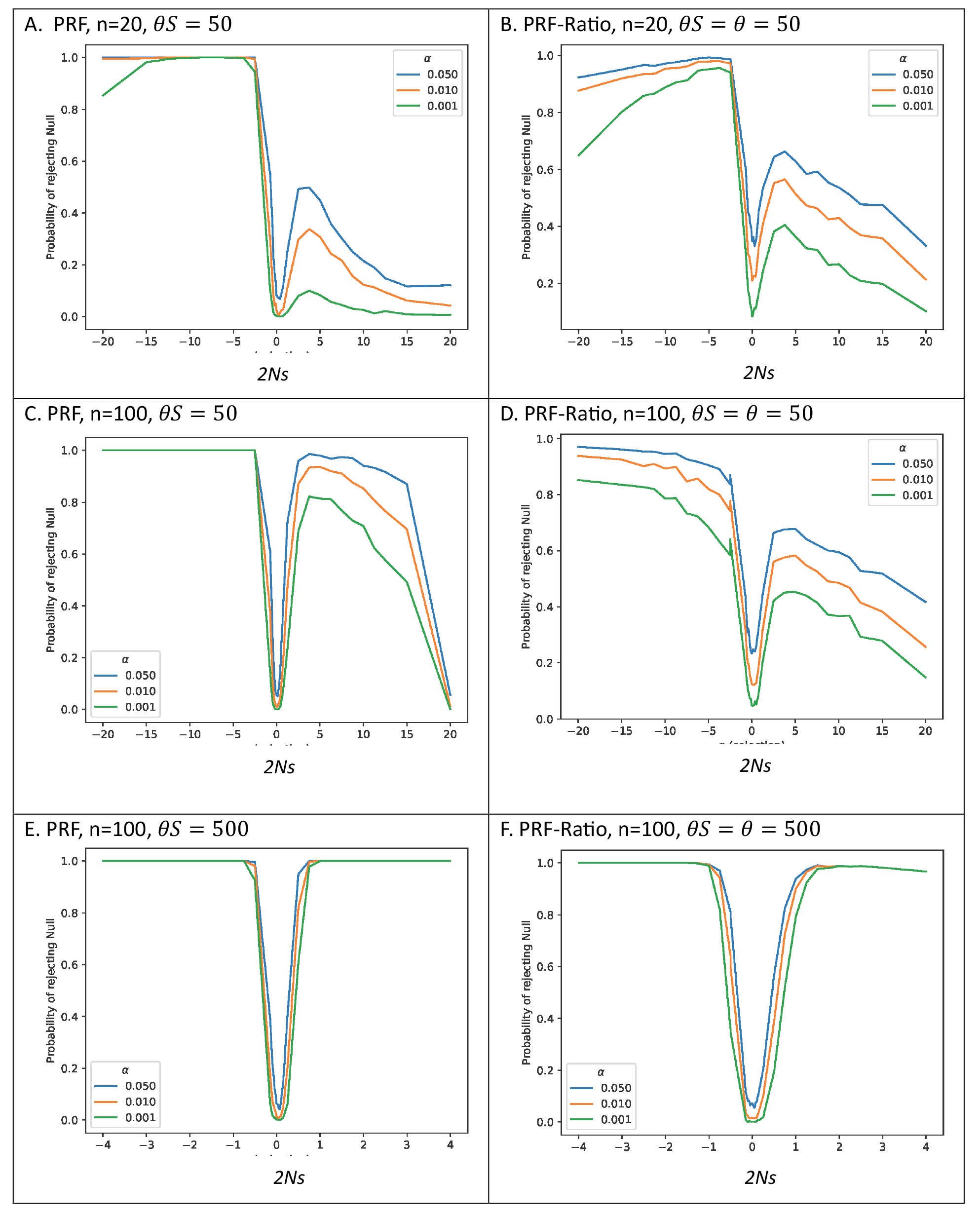

Supplement: S1 Fig — Results for Wright Fisher population Poisson Random Field (PRF) likelihood-ratio tests are shown in panels A, C, and E. Results for PRF-Ratio tests are shown in panels B, D and F. Sample sizes: Few genomes (n = 20) low variation (θ=50) in panels A and B; More genomes (n = 100) and low variation (θ=50) in panels C and D; More genomes (n = 100) and high variation (θ=500) in panels E and F. (TIF) [file pgen.1011427.s001.tif]

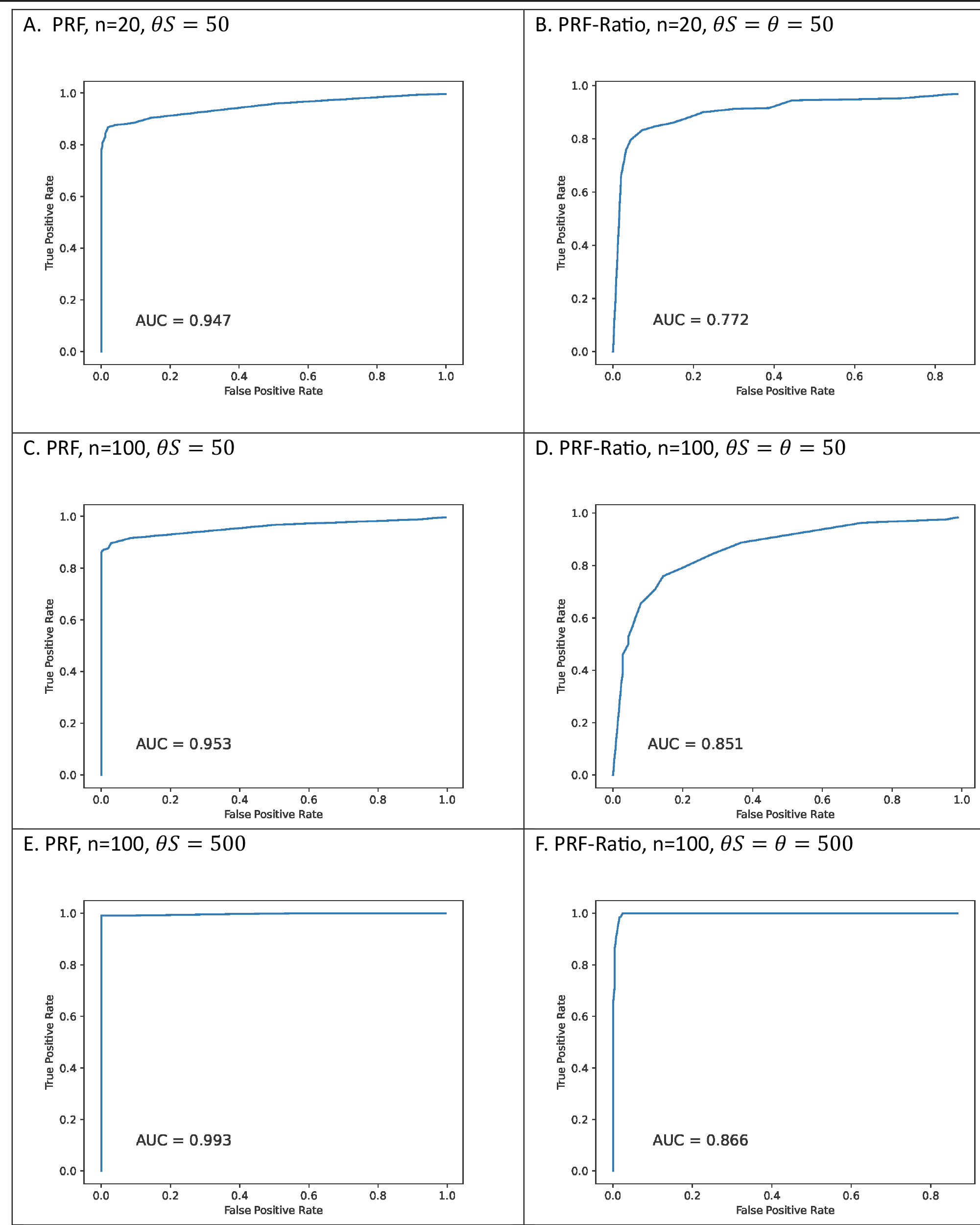

Supplement: S2 Fig — Results for Wright Fisher population Poisson Random Field (PRF) likelihood-ratio tests are shown in panels A, C, and E. Results for PRF-Ratio tests are shown in panels B, D and F. Sample sizes: Few genomes (n = 20) and low variation (θ=50) in panels A and B; More genomes (n = 100) and low variation (θ=50) in panels C and D; More genomes (n = 100) and high variation (θ=500) in panels E and F. (TIF) [file pgen.1011427.s002.tif]

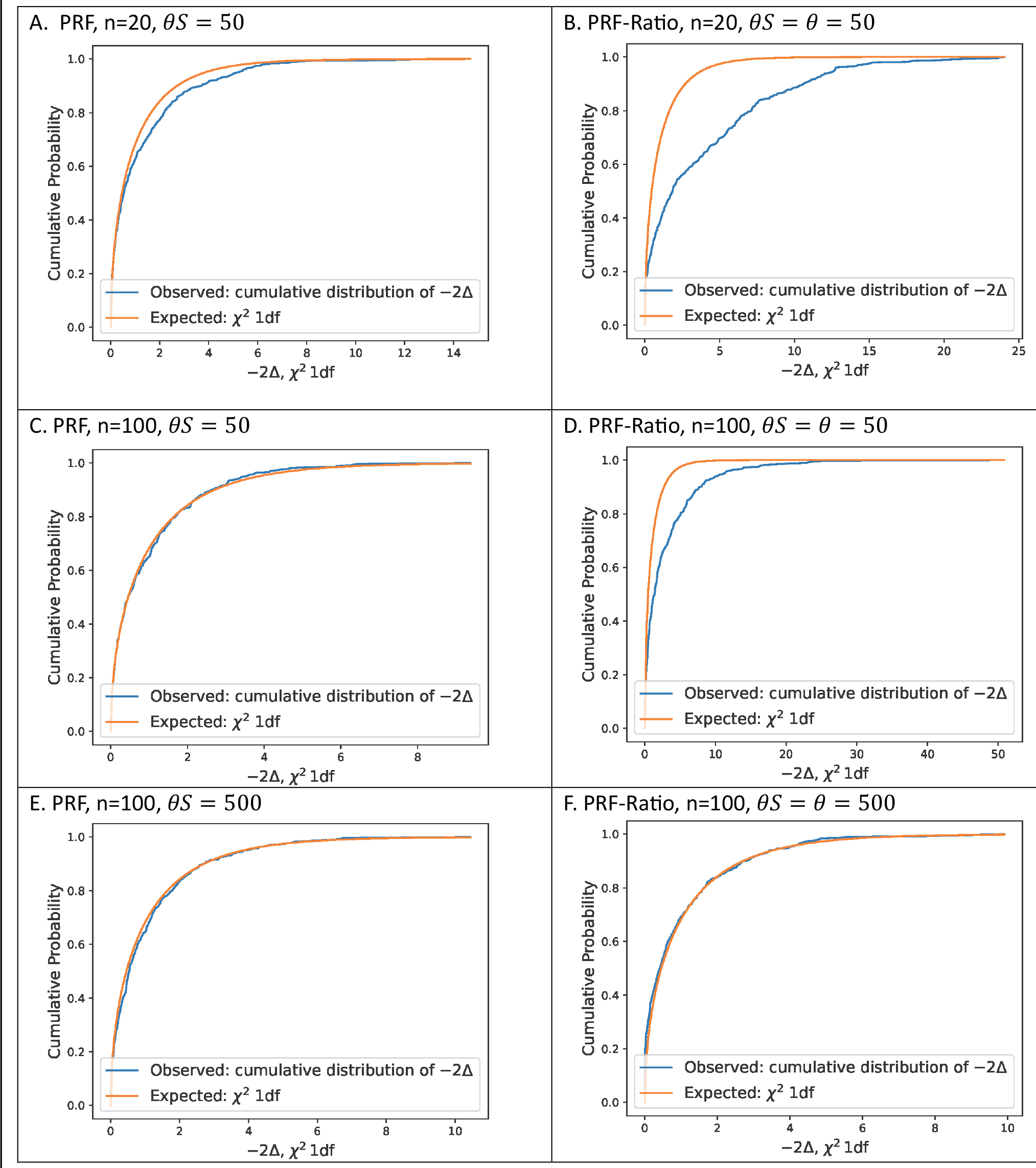

Supplement: S3 Fig — Results for Wright Fisher population Poisson Random Field (PRF) likelihood-ratio tests are shown in panels A, C, and E. Results for PRF-Ratio tests are shown in panels B, D and F. Sample sizes: Few genomes (n = 20) and low variation (θ=50) in panels A and B; More genomes (n = 100) and low variation (θ=50) in panels C and D; More genomes (n = 100) and high variation (θ=500) in panels E and F. (TIF) [file pgen.1011427.s003.tif]

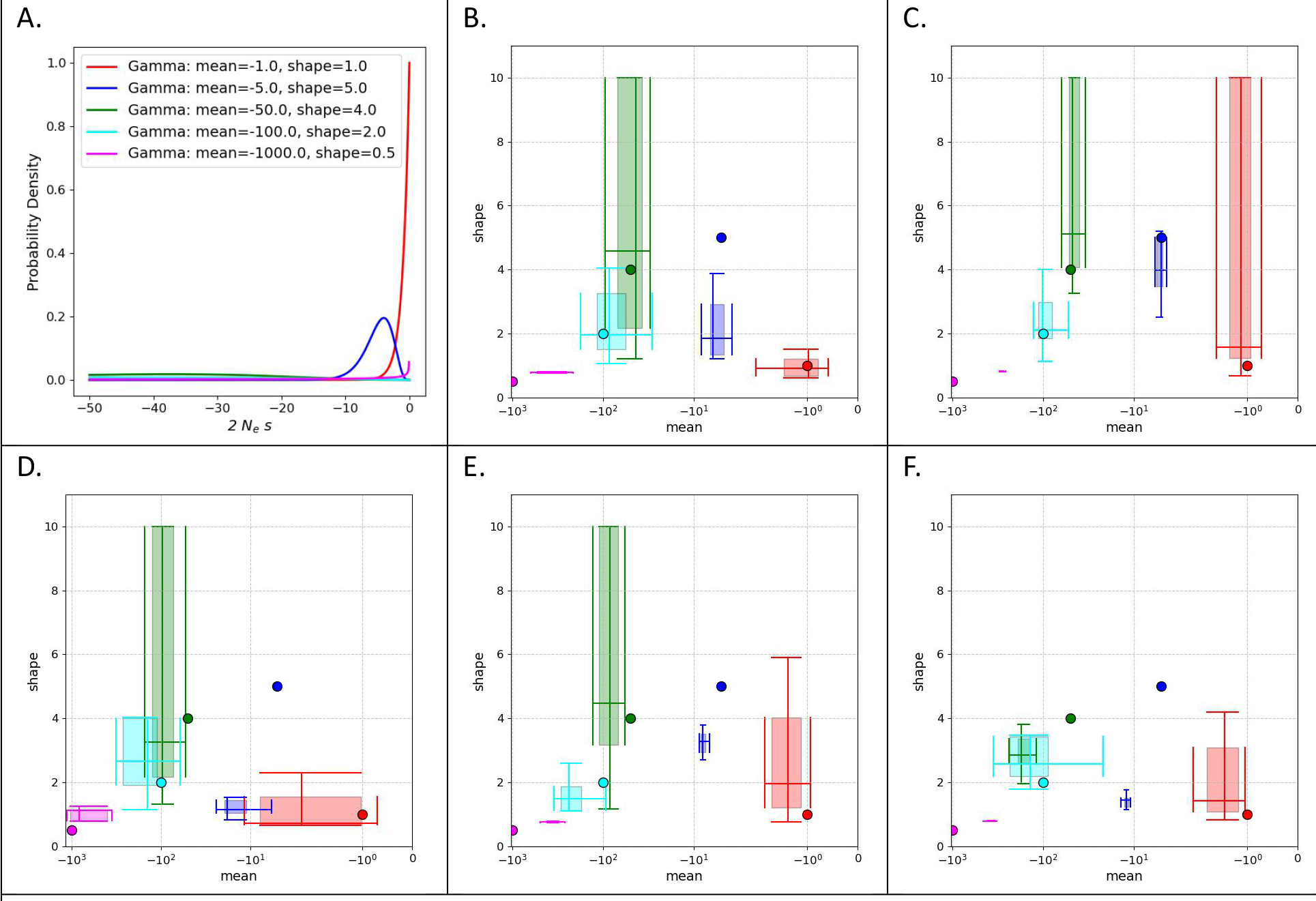

Supplement: S4 Fig — For each simulated data set, γ values were drawn from one of 5 gamma distributions. 20 data sets were simulated for each demographic model and each γ distribution. A. Gamma distributions for random variable x,(0 < x<∞),2Ns=-x. B. Constant population size Wright-Fisher. C. Population expansion. D. Population bottleneck. E. Two populations. F. African Origin model. (TIF) [file pgen.1011427.s004.tif]

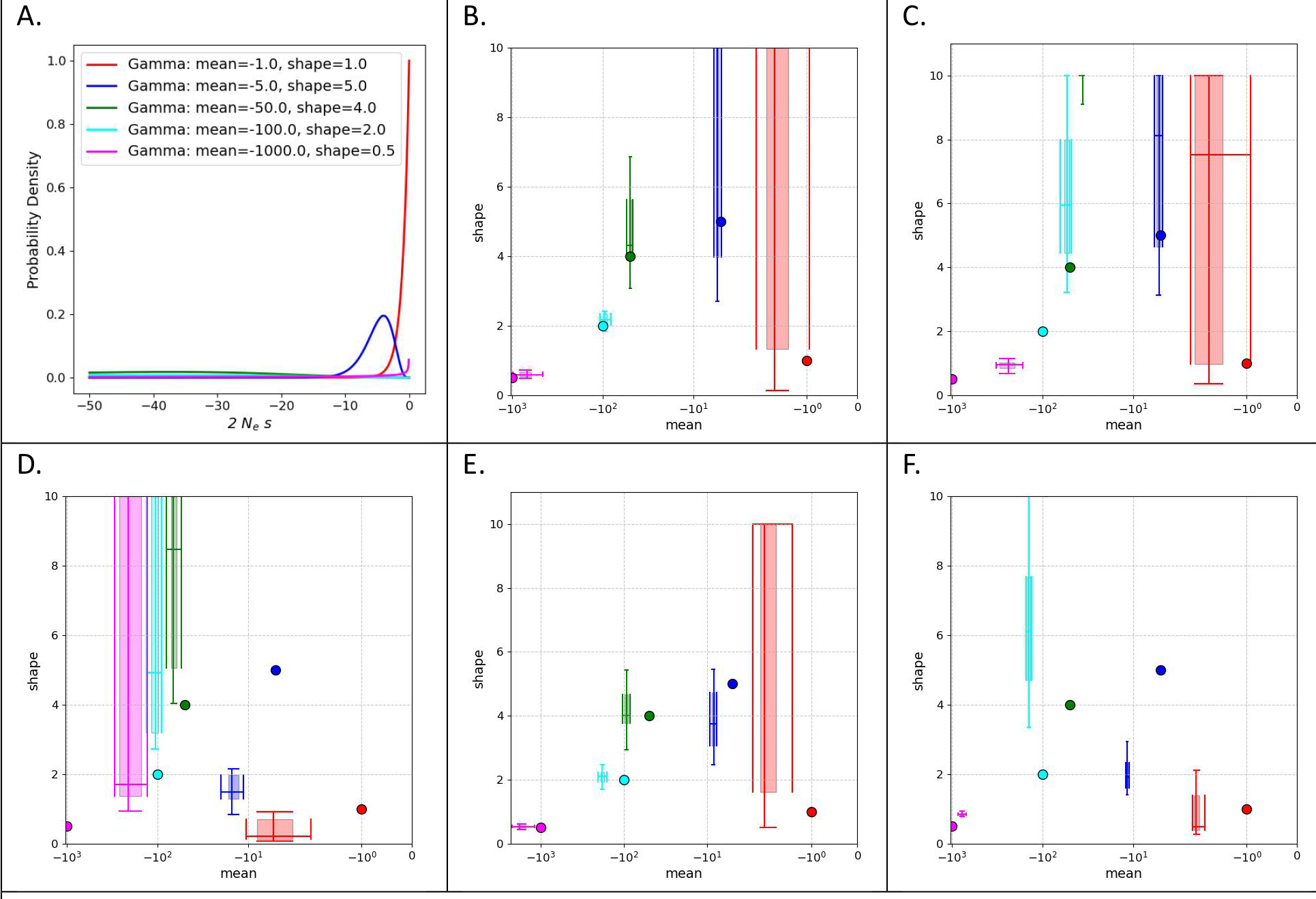

Supplement: S5 Fig — For each simulated data set, γ values were drawn from one of 5 gamma distributions. 20 data sets were simulated for each demographic model and each γ distribution. A. Gamma distributions, for random variable x,(0 < x<∞), 2Ns=-x. B. Constant population size Wright-Fisher. C. Population expansion. D. Population bottleneck. E. Two populations. F. African Origin model. (TIF) [file pgen.1011427.s005.tif]

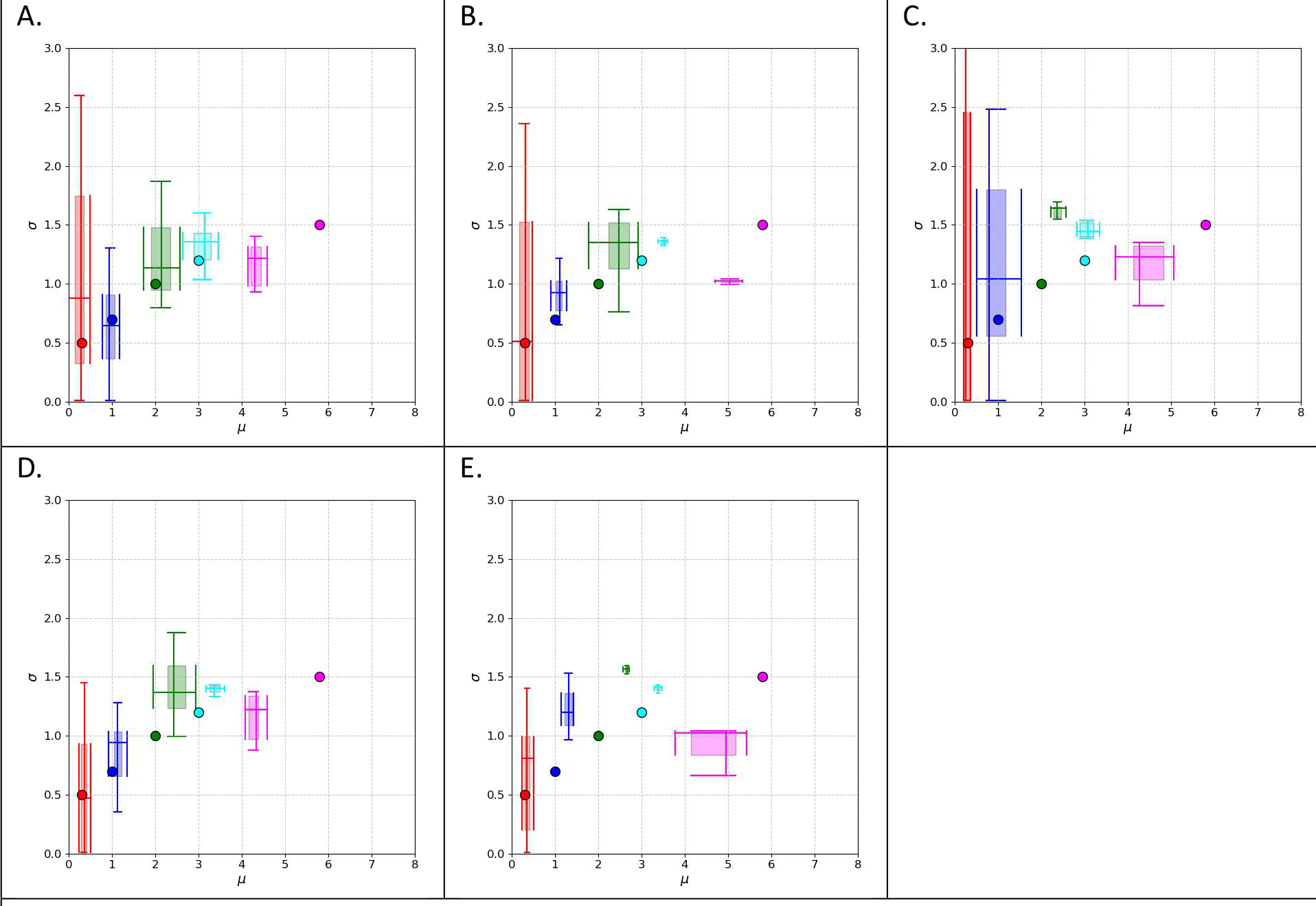

Supplement: S6 Fig — For each simulated data set, γ values were drawn from one of 5 lognormal distributions (see Materials and Methods and Fig 4). 20 data sets were simulated for each demographic model and each γ distribution. A. Constant population size Wright-Fisher. B. Population expansion. C. Population bottleneck. D. Two populations. E. African Origin model. (TIF) [file pgen.1011427.s006.tif]
